# A novel guided surgery system with a sleeveless open frame structure: a retrospective clinical study on 38 partially edentulous patients with 1 year of follow-up

**DOI:** 10.1186/s12903-019-0940-0

**Published:** 2019-11-21

**Authors:** Jaafar Mouhyi, Maurice Albert Salama, Francesco Guido Mangano, Carlo Mangano, Bidzina Margiani, Oleg Admakin

**Affiliations:** 1 Casablanca Oral Rehabilitation Training & Education Center (CORTEC), Casablanca, Morocco; 2 Biomaterials Research Department, International University of Agadir (Universiapolis), 8143 Agadir, Morocco; 3 Department of Periodontics, University of Pennsylvania, Philadelphia, PA USA; 4 Department of Periodontics, Medical College of Georgia, Atlanta, GE USA; 5 Department of Prevention and Communal Dentistry, Sechenov First Moscow State Medical University, 119992 Moscow, Russia; 6 Department of Dental Sciences, Vita and Salute University, San Raffaele, Milan, Italy

**Keywords:** Guided implant surgery, Open-frame sleeveless guides, Fit, Stability, Complications

## Abstract

**Background:**

This retrospective clinical study aims to present results of experience with a novel guided surgery system with a sleeveless, open-frame structure, in which the surgical handpiece (not the drills used for preparation) is guided.

**Methods:**

This study was based on an evaluation of the records of partially edentulous patients who had been treated with a sleeveless open-frame guided surgery system (TWIN-Guide®, 2Ingis, Brussels, Belgium), between January 2015 and December 2017. Inclusion criteria were patients with good systemic/oral health and a minimum follow-up of 1 year. Exclusion criteria were patients who had been treated without a guide, or with a guide with sleeves, patients with systemic/oral diseases and who did not have a follow-up of 1 year. The main outcomes were surgical (fit and stability of the surgical guide, duration of the intervention, implant stability, and any intra-operative or immediate post-operative complication), biologic, and prosthetic.

**Results:**

Thirty-eight patients (24 males, 14 females; mean age 56.5 ± 14.0 years) were included in the study. These patients had been treated with 110 implants inserted by means of 40 sleeveless, open-frame guides. With regard to fit and stability, 34 guides were excellent, 4 acceptable, and 2 inadequate for use. The mean duration of the intervention was 23.7 (± 6.7) minutes. Immediately after placement, 2 fixtures were not stable and had to be removed. Two patients experienced pain/swelling after surgery. The 108 surviving implants were restored with 36 single crowns and 32 fixed partial prostheses (24 two-unit and 8 three-unit bridges); these restorations survived until the 1-year follow-up, with a low incidence of biologic and prosthetic complications.

**Conclusions:**

Within the limits of this study, this novel guided surgery system with sleeveless, open frame–structure guides seems to be clinically reliable; further studies on a larger sample of patients are needed to confirm these outcomes.

## Background

Static guided oral surgery consists of the insertion of dental implants in the exact position, inclination, and depth [1, 2], through the use of customized tooth- [3], bone- [4], or mucosa-supported [4, 5] surgical guides designed with dedicated software and physically realized by three-dimensional (3D) printing [3, 6] or milling [7].

Theoretically, the insertion of dental implants through a surgical guide in an ideal position, planned in the computer, would represent an undoubted advantage for the surgeon [1–6, 8]; it would allow one to reduce the risks related to the invasion of anatomical structures (such as the inferior alveolar nerve and maxillary sinus, or the periodontal ligament and the roots of adjacent teeth, where present) and to obtain an ideal prosthetic emergence through the preparation of a virtual 3D diagnostic wax-up [8, 9]. This 3D wax-up, in fact, realized on a model captured by intraoral [10] or desktop [11] scanning within computer-assisted-design (CAD) software and imported into the guided surgery software, guides the insertion of the fixtures in the exact position and inclination, facilitating the prosthetic rehabilitation process [8, 9, 12]. Last but not least, guided surgery allows insertion without the need to raise a mucoperiosteal flap [13], which allows one to reduce the post-operative pain of the patient and the duration of the intervention [14, 15].

Despite these indisputable advantages, however, only a limited number of clinicians routinely use guided implant surgery today, and then almost exclusively for implant placement in completely edentulous patients [2, 9, 16, 17]. The causes of this are different, and only partly attributable to the costs of the design and fabrication of the surgical guide (often performed by external services). Of course, the planning process takes time, and a learning curve is necessary for the clinician to learn how to design with software [8, 18]; moreover, the costs of the required machines (cone beam computed tomography [CBCT] [19], intraoral or desktop scanner [6, 20], and, if the clinician wants to produce the guides, 3D printer [21]) can be quite high. But maybe these are not the real reasons why guided surgery has still not spread universally in the dental world, particularly, in the implant-supported restoration of partially edentulous patients [6, 18, 22].

The real reason why guided implant surgery in the partially edentulous patient has not yet spread globally could lie with the design of surgical templates [6, 23]. In fact, starting from 1992, when the concept of guided implant insertion was introduced, the design of the surgical guides proposed by the various software manufacturers or implant companies remained substantially the same and did not evolve [6, 23]. Most of the tooth-supported surgical guides currently available still remain resin bites with extended surfaces, which rest on the adjacent teeth, completely covering the area below; the preparation of the implant site and the implant positioning are still carried out by means of a metal sleeve positioned inside the template, in which the surgeon inserts diameter reducers [2, 4, 6, 24].

This conventional approach presents various clinical problems. Firstly, in the posterior maxilla and mandible of partially dentate patients, the components necessary to insert implants in a guided manner (long preparation drills, surgical template, and sleeves) often steal too much space and thus do not allow the clinician to work [2, 6, 9, 25]. The presence of the sleeve, in fact, forces the surgeon to use long drills, available only in surgical kits specifically dedicated to guided surgery: only with these long drills, in fact, it is possible to prepare the implant site at the correct depth. Unfortunately, the limited opening of the patient’s mouth and the presence of teeth in the antagonist arch do not allow the insertion in situ of the necessary components; hence, proceeding with the intervention is impossible [6, 25]. This obviously does not apply to completely edentulous patients [4, 5, 14, 16, 17]. There are, however, other problems such as the lack of fit and stability of the tooth-supported surgical guides, which, once positioned, often tend to move, forcing the clinician and the assistant to hold them in place with their hands [2, 3, 6, 18, 21, 22]. The lack of stability is a danger, since it can determine spatial deviations in the insertion of the implants, compared to the original planning [2, 3, 6, 18, 21, 22]. Such deviations do not necessarily lead to invasion of dangerous anatomical structures, but may result in an implant placement that is too buccal, which may lead to complications and aesthetic sequelae [3, 6, 18, 21, 22, 26], or insertion too close to other teeth or implants. All these situations can complicate the prosthetic rehabilitation, forcing the dental technician to adopt compromise solutions. The scientific literature has amply reported through systematic reviews [9, 16] that guided surgery is rather inaccurate, with deviations between the planned and the real (actual) position of the implants. The lack of stability of the template depends mainly on its design (and, obviously, the acquisition and prototyping tools used to fabricate it) [21, 24]; the material used may have a role too. In any case, the conventional templates covering the whole dentate arch do not allow the surgeon to have adequate visibility of the operative field (for example, they do not allow him to raise a flap to preserve the keratinized mucosa, which plays an important role in peri-implant health over time) [6]. Moreover, with these conventional templates it may be difficult to irrigate, with the risk of overheating the implant site [6]. Finally, the positioning of the fixture through the metallic sleeve can entail the risk of contamination of the implant surface, with possible negative consequences [27–29].

All these limitations are related to the approach conventionally used in guided surgery, which involves guiding the drills during site preparation through the use of sleeves. But today, there are alternatives to this approach.

The aim of this retrospective clinical study was to present a novel guided surgery system with a sleeveless, open-frame structure. In this system, no sleeves are used; the surgical handpiece is guided, not the drills used for preparation.

## Methods

### Study design; inclusion and exclusion criteria

The present retrospective study was based on the analysis and evaluation of the records of patients who underwent a guided implant surgery procedure, in two clinical centers, in the period between January 2015 and December 2017. Patient records were standardized and contained a whole range of information, such as gender and age at the time of surgery, oral and systemic health status, presence of smoking and/or alcohol use/abuse, type and number of fixtures inserted, their position and characteristics (length and diameter), and type of prosthetic restoration that was subsequently loaded on (single crowns [SC] or fixed partial prosthesis [FPP]).

The records were accompanied by medical imaging, and any complications or problems registered during the guided surgery (poor fit and lack of stability of the template; impossibility of using the template in the posterior sectors for lack of space; fracture of the template; aberrant placement of the implant with or without invasion of anatomical structures) were appropriately noted.

In addition, patient records contained additional information collected during the annual follow-up controls (1/2 per year for each patient, corresponding to planned professional oral hygiene sessions) or subsequent visits, such as the onset of complications or failures and/or the need for corrective actions.

Inclusion criteria for this study were partially edentulous patients treated with a new guided surgery system (TWIN-Guide®, 2Ingis, Brussels, Belgium) based on open-frame and sleeveless templates (where the handpiece was guided, but not the drill). These patients had to be in good oral and systemic health, and they had agreed to return to the clinical centre for control visits and annual professional hygiene sessions. Finally, in order to be included in the study, patients had to be followed for a minimum period of 1 year after surgery.

On the contrary, all patients who were treated with implant insertion without the use of surgical guide or with other conventional surgical templates that foresee the use of sleeves, patients who had oral or systemic diseases and patients who did not have a follow-up of at least 1 year were excluded from the study.

All patients had been treated after having received detailed explanations regarding the procedures to which they were to be subjected and after having accepted them by signing an informed consent; all patients were also informed about enrollment in this retrospective clinical study and consented to analysis of their medical records. Thus, this study respected the principles of the protection of patient health set forth in the Helsinki Declaration on experimentation on human subjects (2008 revision) and was endorsed by the local Ethics Committee of the Sechenov University of Moscow.

### Surgical planning

For each patient, an impression with polyvinyl-siloxane material was taken with a proprietary acrylic radiotransparent tray (2Ingis® tray). This tray incorporated one or more Lego® bricks (Lego®, Copenhagen, Denmark), attached on the external surface of the tray. Before removing the impression tray, the patient underwent CBCT examination. The CBCT was immediately examined by the clinician, in order to verify the available bone volume and thus the feasibility of the surgery. The impression tray was removed and, from this, a stone cast model was poured. After the patient left the dental office, the clinician extracted from the CBCT all digital imaging and communication in dentistry (DICOM) files and sent these data to the 2Ingis® center for a second check of the quality of the CBCT. The DICOM files were imported in the SMOP® software (Swissmeda, Baar, Zurich, Switzerland), where any possible distortion that occurred during the CBCT (from movements of the patient’s head) was investigated, through the superimposition of the radiographic representation of the brick on the original drawing of it (present in the software).

When the correspondence between the radiographic imaging and the original drawing of the Lego® brick was verified, and no distortions occurred, the surgical plan could proceed. The clinician used a desktop scanner to acquire the 3D anatomy of the stone cast model of the patient as well as the impression tray. Then, all these data were sent by email to the 2Ingis® center. The 2Ingis® center then imported the dentate model into the aforementioned planning software and superimposed it on the bone model derived from the CBCT; a careful superimposition was then made, first by points and then by surfaces. Once again, the Lego bricks were useful for the control of the quality of the superimposition, particularly in the presence of scattering/metallic artifacts in the CBCT (derived by the presence of metal-ceramic restorations in the patient’s mouth). Inside the SMOP® planning software, a virtual wax-up was imported or created; then the implants were virtually planned in the exact position, depth, and inclination, taking into account the amount of available bone as well as the prosthetic emergence profile. The planning took place within the SMOP®, a planning software in which the conventional sleeves are present. However, the planning started from a different concept: not from the length of the implant, but from the length of the drills available for preparation. In detail, a “zero point” was obtained, as the sum of the distance between the sleeve and the implant shoulder, plus the height of the sleeve, plus a fixed value set at 12 mm (ISO value). Therefore, a “depth value” was obtained, by the subtraction between the length of the available preparation drill(s), minus the “zero point” value. The depth value had to correspond to the planned implant length: if this correspondence was present, no spacers were needed during surgery. Conversely, if the depth value was greater than the planned implant length, the use of spacers was needed during surgery. The surgical planning was thus completed and shared between the 2Ingis® center and the clinician for final check, improvements, and approval. Once the implant planning was approved, the engineers of the TWIN-Guide® center designed the open-frame, sleeveless surgical guide, using a proprietary software (2Ingis CAD software®), according to the established plan. The peculiar characteristic of these guides was the presence of an open structure, with selective supports on the adjacent teeth. Moreover, these open templates did not have the classic holes for inserting metal sleeves and/or reducers, conventionally used for guiding the preparation drills; it was instead the surgical handpiece to be guided, by means of a proprietary adapter characterized by two full cylinders (male), which were inserted in two hollow cylinders (female) incorporated into the guide and placed externally and internally to the residual bone crest (not at the above it). Basically, the drill was free from any interference, but the handpiece was double-guided. These open-frame, sleeveless surgical guides were then manufactured either in metal, using an industrial laser-sintering machine (Pro-X DMP200®, 3D Systems, Rock Hill, SC, USA), or in resin, using a powerful 3D printer (Nextdent®, Vertex Dental, Soesterberg, The Netherlands). The guides were then sterilized and sent to the dental office.

### Surgical and prosthetic procedures

Before starting the operation, the patients were asked to rinse with a chlorhexidine-based mouthwash 0.2% for at least 4 min. Infiltration anesthesia followed, with articaine with adrenaline (1:100,000), then the template was positioned. The fit and stability of the surgical guide were carefully checked at this stage. The support of the template was made by points (not by surfaces), on the adjacent teeth, and fit and stability had to be sufficient to allow the surgeon to proceed with the operation. If the fit or the support were unsatisfactory, the surgeon could not proceed with the intervention and was forced to proceed in a conventional manner (not guided), through the elevation of a mucoperiosteal and manual preparation/positioning of the implants. If instead the fit and the stability were satisfactory, the intervention proceeded with the passage of a mucotome, for the removal of the mucosal operculum and for accessing the underlying bone plane (flapless technique). However, where the surgeon believed it was necessary to preserve the keratinized tissue, a small crestal incision (without releasing incisions) was performed, in order to keep the keratinized mucosa, moving it buccally to the implant site. The preparation of the surgical site then proceeded, in full accordance with the indications of the implant house, through the passage of a leveling drill, one drill for the depth and the subsequent ones with incremental diameters. All these steps took place with the surgeon having visibility of the operative field, and under abundant physiological irrigation. Once the depth and, above all, the adequate size of the preparation was reached, the implants were inserted into the prepared sites, again using the guide. The surgeon placed the implant of a length and diameter corresponding to the original 3D surgical planning. The implant was initially inserted through the handpiece, set with a maximum insertion torque of 35 Ncm; exceeding this threshold, the surgeon proceeded manually for better control. When the implant was placed, the surgeon proceeded to remove the template and, where needed, sutured. When an immediate restoration was needed, as in the anterior areas or in the case of one-piece implants, a temporary shell in acrylic resin was relined chairside on the abutment and delivered immediately after surgery. Alternatively, a polyvinylsiloxane impression was captured, and the provisional preparation was done in the laboratory. The temporary restoration was delivered within 48 h of surgery and cemented with a zinc-oxide eugenol cement (TempBond®, Kerr, Orange, CA, USA). In all cases, before cementation, the restorations were carefully polished to obtain an ideal emergence profile. The occlusion was meticulously controlled so as to avoid pre-contacts, in both protrusion and laterality. An intraoral periapical radiograph was obtained with the temporary restoration in position and, after that, the patient could be discharged with analgesics and antibiotic prescription (600 mg ibuprophene every 12 h, for 2 days, and amoxicillin + clavulanic acid, 2 g per day, for 6 days). Conversely, when a delayed prosthetic protocol was selected, the impressions for the provisional restorations were scheduled 1 or 2 months after the surgery. In all cases, the first follow-up visit was set at 10 days after the intervention. The temporaries remained in situ for a period of 2 months; after which they were replaced with the definitive metal-ceramic or zirconia restorations. In the latter case, translucent zirconia (Katana®, Kuraray Noritake, Tokyo, Japan) was employed. In all cases, the final restorations were cemented with zinc-oxide eugenol cement. Before cementation, occlusion was carefully checked with articulating papers (Bausch Articulating Paper®, Bausch Inc., Nashua, NH, USA) in order to avoid any static/dynamic precontact. After cementation, another intraoral periapical radiograph was obtained. The patient was then enrolled in a recall program, for professional oral hygiene sessions every 6 months.

### Study outcomes

Each of the patients included in the study was followed for at least 1 year after implant placement, through 1 to 2 annual check-ups for professional oral hygiene sessions. The outcomes of the present study were of a surgical nature (i.e., linked to the execution of the intervention and the 2-week period immediately after) and of a biologic and functional nature (i.e., linked to the possible biologic and prosthetic complications that could occur to the implant-supported restorations during the 1-year follow up).

In detail, the main study outcomes were as follows:

1. Surgical outcomes (related to the guided surgery procedure)

- fit of the surgical guide
- stability of the surgical guide
- duration of the intervention
- intra-operative and immediate post-operative complications
- implant stability at placement

2. Biologic outcomes

- presence/absence of peri-implant mucositis
- presence/absence of peri-implantitis

3. Prosthetic outcomes

- presence/absence of mechanical complications
- presence/absence of technical complications

### Surgical outcomes

#### Fit of the surgical guide

The fit of the surgical guide represented one of the primary outcomes of the present study, and consisted of the template’s ability to adapt perfectly to the pre-defined support points, without open spaces (gaps) and at the same time without pointing above the teeth. By definition, the fit of the template could be defined as excellent (if perfect, without any gap or interference), acceptable (if sufficient, with minimal interference that still allowed an adaptation in post-processing, in the laboratory, through polishing), or inadequate. The fit test was performed before starting the surgery and consisted of a careful inspection analysis of the adaptation of the template on the occlusal surfaces of the supporting teeth, in the different sections. The correspondence and the contact between the surface of the template and the supporting teeth had to be perfect, at the occlusal level, but the fit of the template also depended on the adaptation on the approximal (mesial and distal) surfaces of the adjacent teeth, and on the perfect adhesion to their buccal and palatal (lingual) contacts. After this careful visual inspection, the surgeon could define the fit of the template as excellent, acceptable, or inadequate. If it was excellent, the clinician could proceed to verify the stability of the template. If it was acceptable, and therefore required some retouching, the clinician could adapt the guide through polishing in the laboratory and then retest the fit in the mouth. In any case, these adaptations had to be minimal, in order not to compromise the correct insertion of the fixture, according to the position, inclination, and depth planned in the software. Finally, if the fit was completely unsatisfactory, the clinician could not proceed with the guided surgery and therefore had to insert the implants manually, according to conventional protocols; in the latter case, guided surgery was considered a failure.

#### Stability of the surgical guide

As for the fit, the stability of the surgical guide was verified by the clinician at the time of surgery. A surgical template was defined as stable in a case in which, besides possessing a perfect adaptation, it was immobile during all the phases of the surgery (preparation of the surgical site with drills of incremental diameter, and implant insertion). Stability was defined as excellent if the template did not move at all during the operation, exerting some resistance to insertion and removal. Stability was defined as acceptable if the template had a minimal, negligible swinging / jiggling movement during the preparation of the implant site, forcing the surgeon to keep it in place manually. But if, instead, the movement were not manageable, the template was defined as unstable and could not be used; the surgeon therefore had to proceed to raise a full-thickness flap and prepare the implant site manually, in a conventional manner. In the latter case the guided surgery procedure was considered a failure. In all cases, as was the case with the fit, the stability of the surgical guide was reported in the patient’s medical record.

#### Duration of the intervention

The chair assistant monitored exactly the time required for surgery, from the anesthesia to the insertion of the implant and the final removal of the surgical guide. The time was measured in minutes and noted in the patient’s folder. The mean time per implant was then calculated, by dividing the overall time required for the surgical procedure by the number of fixtures inserted.

#### Intra-operative and immediate post-operative complications

Any complications occurring during the operation were noted in the patient’s file and were reported among the results of the present study. Among the intra-operative complications were: fracture of the surgical guide, inadequate opening of the mouth by the patient (which made the procedure impossible), insertion of the implant in aberrant position/ inclination/ depth, compared to the plan provided in the guided software, with perforation of one of the corticals (buccal or palatal/ lingual), invasion of noble and insurmountable anatomical structures (inferior alveolar nerve, maxillary sinus, periodontal ligament of adjacent teeth), which required the opening of a full-thickness flap and the immediate removal of the implant.

Conversely, the immediate post-operative complications were the complications that could occur in the 2 weeks following the surgery. They included pain, discomfort, exudation and suppuration, swelling, and infection of the implant.

#### Implant stability at placement

The stability of each fixture was checked clinically, immediately after placement, by applying a reverse torque of 20 Ncm [30].

### Biologic outcomes

All the biologic complications that could affect the implants from the second week of surgery until the end of the study were marked in the patient’s record. These complications included peri-implant mucositis and peri-implantitis. The threshold for defining peri-implantitis was set at a probing pocket depth ≥ 6 mm, with bleeding/ suppuration on probing and evidence of peri-implant bone loss > 3.0 mm [31].

### Prosthetic outcomes

All the prosthetic complications that could affect the implants from the second week of surgery until the end of the study were marked in the patient’s record. These complications included mechanical complications, such as screw loosening and/or fracture [32], as well as technical complications, such as ceramic chipping/ fractures or fractures of the metal framework of the restorations [33].

### Statistical evaluation

All data were extracted from the individual patient records by an independent operator, not directly involved in the insertion of the implants and their prosthesis, at the end of the 1-year follow-up period. The descriptive statistical analysis included the description of the demographic characteristics of the patients (gender, age at the time of surgery, smoking habit) and the characteristics of the implants inserted (brand, site, position, length, and diameter) and restorations placed (SC and FPP). A Pearson Chi Square test was used to analyze homogeneity in the patient and implant distribution. Absolute and relative frequency (%) distributions were calculated for qualitative variables (fit and stability of the surgical templates, intra-operative and immediate post-operative complications, implant stability) while means, standard deviations (SD), medians, and confidence intervals (95%CI) were estimated for quantitative variables (patient’s age at surgery, duration/time of the surgery). Implant stability, survival, and the incidence of complications were calculated at the restoration level.

## Results

In total, 38 patients (24 males and 14 females) between 20 and 80 years of age (mean age 56.5 ± 14.0 years; median 59.5; 95%CI: 52.1–60.9) were included in the present retrospective study. A summary of the patients’ characteristics is provided in Table 1. These patients had been treated with 110 fixtures (38 Megagen®^,^ Gyeongbuk, South Korea; 53 Dentium®^,^ Cypress, CA, USA; and 19 implants from other brands) inserted by means of 40 sleeveless, open-frame surgical guides. Among the guides, 25 were fabricated in metal, and 15 were in resin. A summary of the implants’ features is provided in Table 2. Among the fixtures, 55 were inserted without the elevation of any surgical flap. According to the pre-established planning, 36 implants had to be restored with SCs and 74 implants had to be restored with FPPs. Among the surgical guides, 34 (85%) had excellent fit and stability, 4 (10%) had acceptable fit and stability, and only 2 (5%) had inadequate fit and stability for clinical use. The two guides with inadequate fit and stability were made in resin. The mean duration of the intervention was 23.7 min (± 6.7, median 22, 95%CI: 21.7–25.7) per template, which resulted in a mean time per implant of 6.5 min. No immediate intra-operative complications were reported: no fracture of the surgical guide occurred, and all patients had a sufficient mouth opening to allow the surgeon to proceed with surgery. No implants were placed in an aberrant position/inclination/depth, no perforations of the corticals was evidenced, nor invasion of any anatomical invalicable structure (inferior alveolar nerve, maxillary sinus, periodontal ligament of adjacent teeth). However, two Dentium® fixtures (1.8%) were not stable at placement and consequently had to be removed. In addition, in the immediate post-operative period, two patients (5.2%) suffered pain and swelling; these patients were prescribed additional oral analgesics. The 108 surviving implants were restored with 36 SCs and 32 FPPs (24 two-unit bridges and 8 three-units bridges, respectively). These restorations survived for the entire 1-year follow-up time, without any implant failure/removal registered. Among the biologic and prosthetic complications registered during the follow-up, however, there were two instances of peri-implant mucositis (1.8%), two abutment screw loosenings (2.9%) (in two SCs), and one ceramic chipping/fracture (1.4%) (in a three-unit FPP).

**Table. 1:** Patient demographics

Patient characteristics	n° of patients	*p* value^*^
Gender		
Males	24	.247
Females	14	
Age at surgery		
20–35 years	2	.104
36–50 years	11	
51–65 years	15	
66–80 years	10	
Smoking habit		
No	25	.163
Yes	13	
Total	38	–

^*^ Pearson’s Chi square test

**Table 2:** Implant distribution

Implant features	n° of implants	*p* value^*^
Implant brand		
Megagen®	38	.014
Dentium®	53	
Others	19	
Implant site		
Maxilla	65	.175
Mandible	45	
Implant position		
Incisors	15	.075
Cuspids	22	
Premolars	34	
Molars	39	
Implant length		
< 10 mm	33	< 0.001
10–12 mm	71	
> 12 mm	6	
Implant diameter		
< 4 mm	34	< 0.001
4–5 mm	68	
> 5 mm	8	
Prosthetic restoration		
Single crown (SC)	36	.009
Fixed partial prosthesis (FPP)	74	
Total	110	–

^*^Pearson’s Chi square test

In Figs. 1, 2, 3, 4, 5, 6, 7, 8, 9, 10, 11 and 12, one case of anterior implants with a metal guide is fully documented in all phases. In Figs. 13, 14, 15, 16 and 17, a complete case of posterior implants with a resin guide is documented in the main phases.

**Fig. 1:**
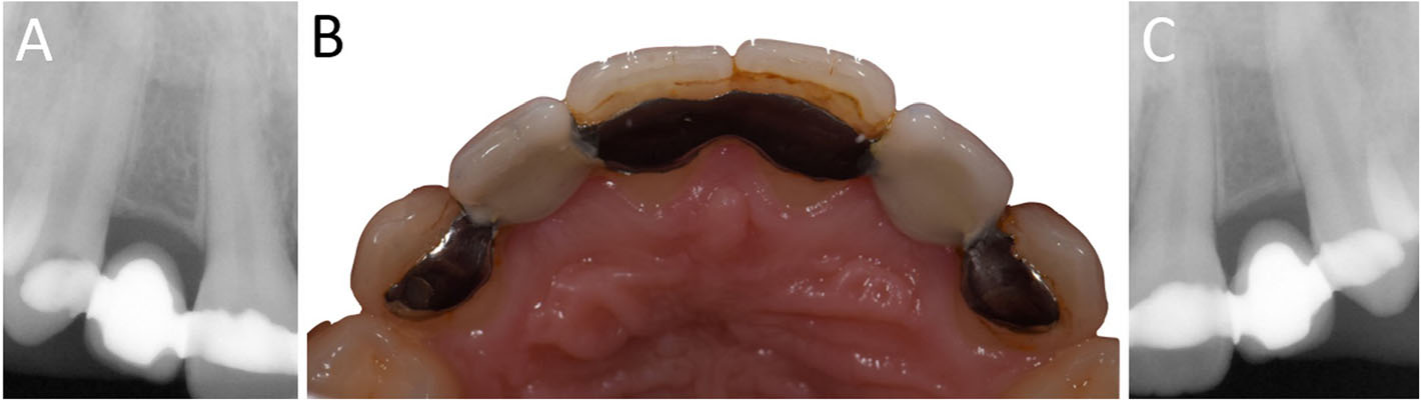
Pre-operative situation. The young patient presented with an old Maryland bridge, and asked the surgeon to replace it with two fixed implant-supported restorations. **a** Right side, radiographic control. The horizontal space between the roots of the adjacent teeth was narrow. **b** The Maryland bridge in position, occlusal view.; **c** Left side, radiographic control. The horizontal space between the roots of the adjacent teeth was narrow

**Fig. 2:**
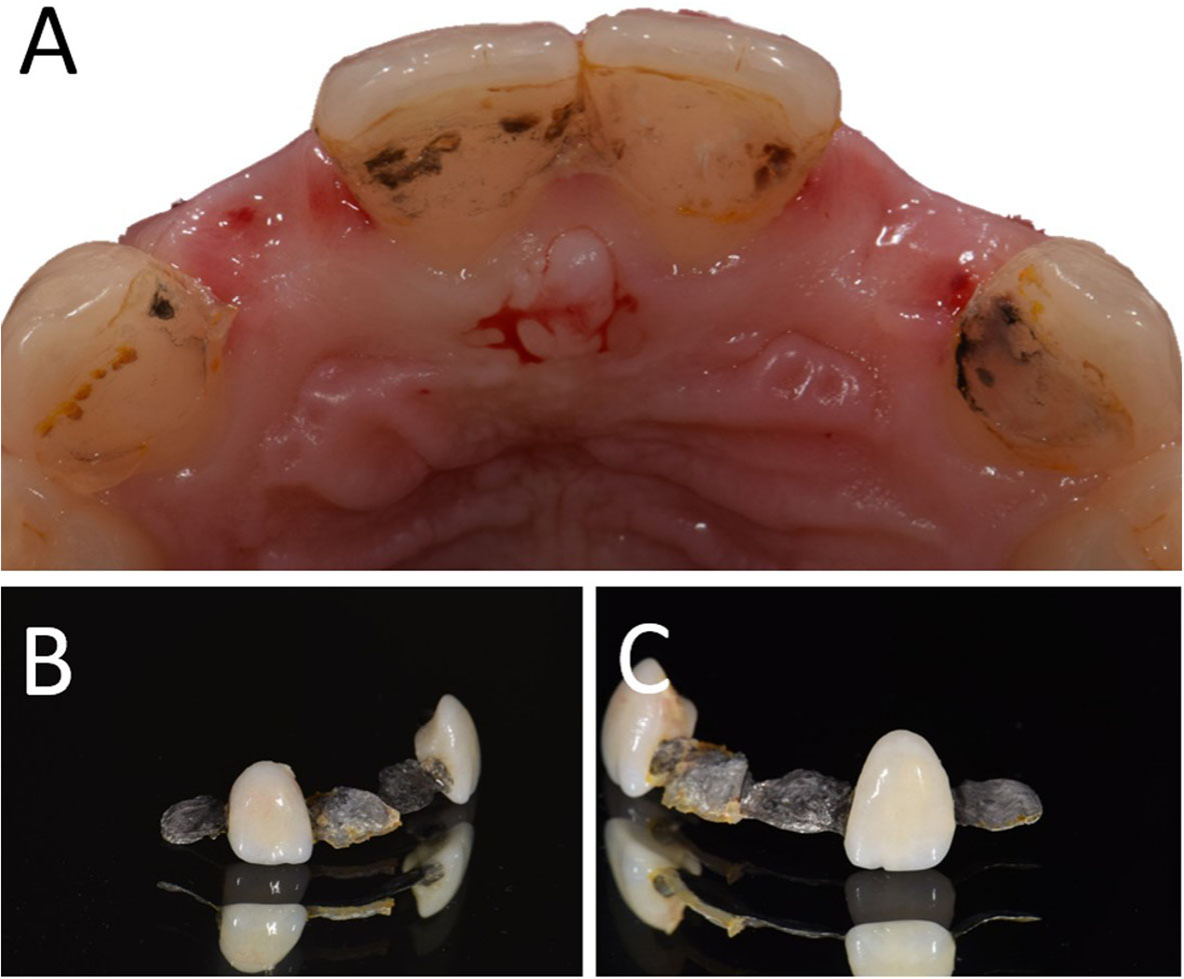
Pre-operative situation. Removal of the Maryland bridge. **a** Occlusal view; **b** Details of the Maryland bridge after removal, right side; **c** Details of the Maryland bridge after removal, left side

**Fig. 3:**
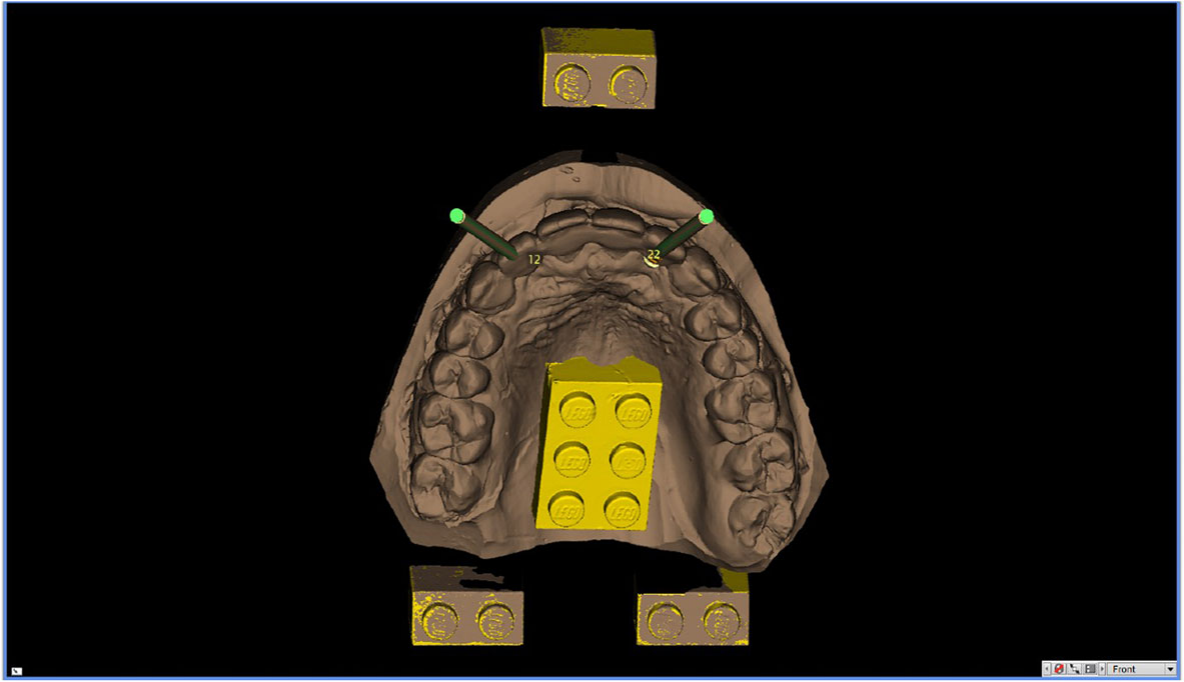
The model of the teeth is imported in the guided surgery software and superimposed on the CBCT reconstruction by means of reference points (Lego bricks). These reference points are also useful to understand the quality of the CBCT, in order to highlight any possible patient movement and possible related CBCT distorsion. The superimposition, by points and surfaces, is extremely accurate

**Fig. 4:**
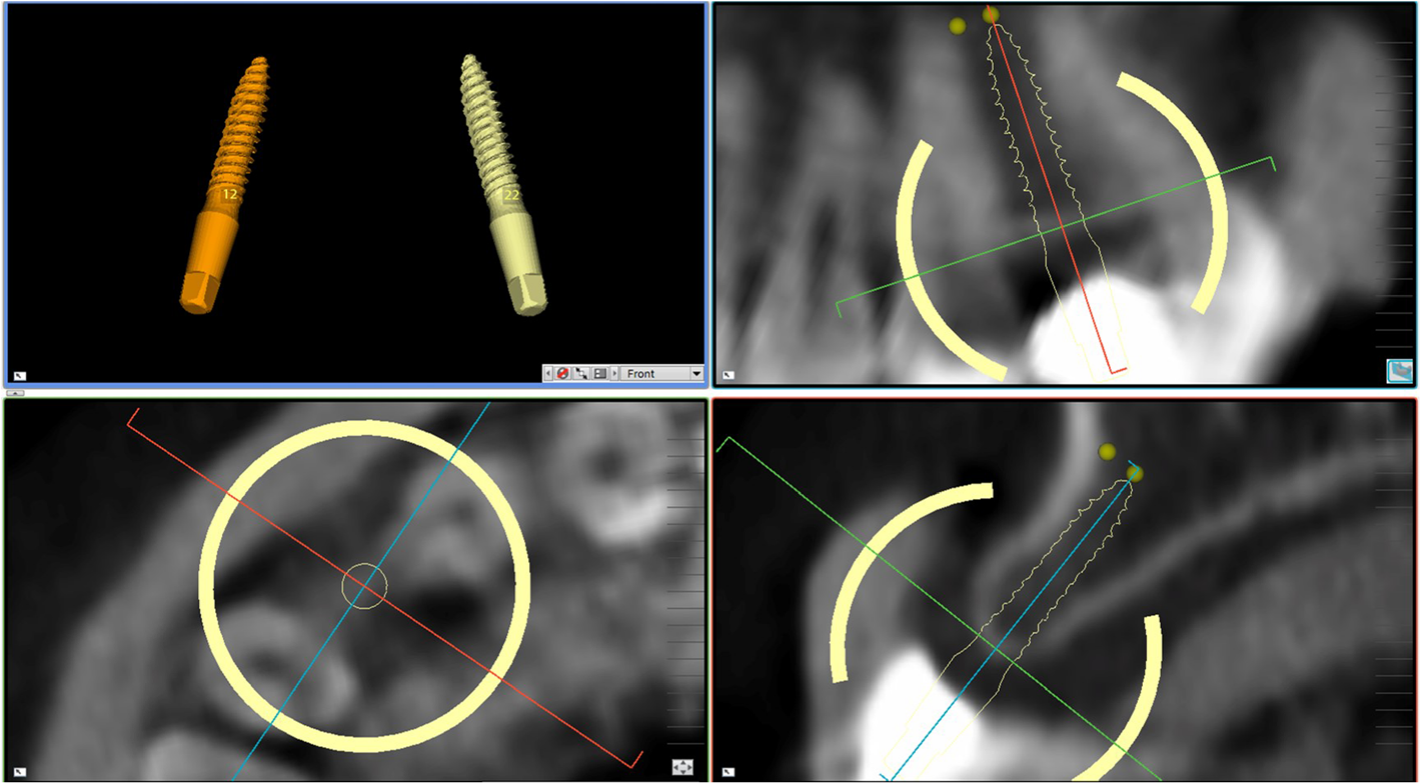
Planning of implant placement with the 2Ingis® guided surgery software. The position, inclination and depth of the right maxillary incisor is carefully planned, in order not to collide with the roots of the adjacent teeth

**Fig. 5:**
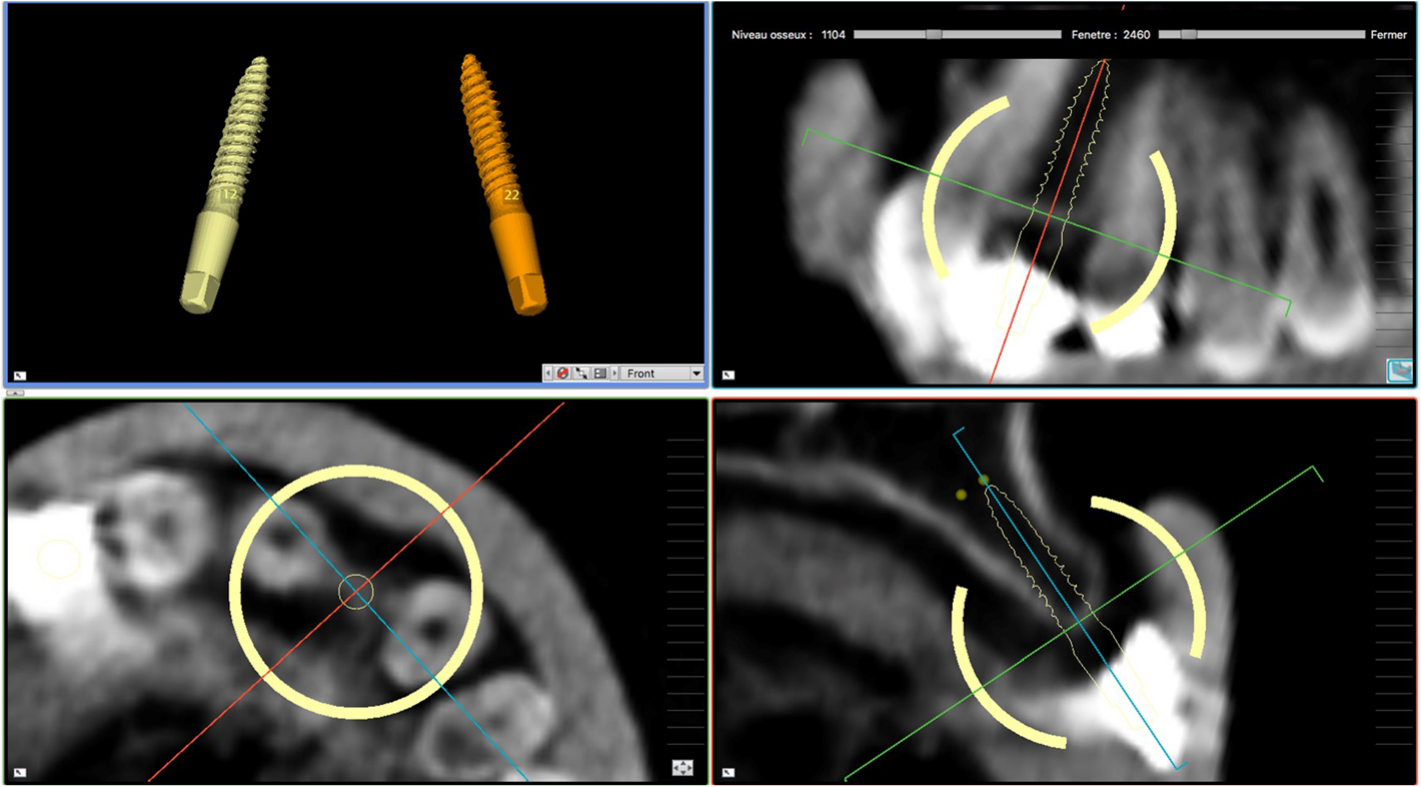
Planning of implant placement with the 2Ingis® guided surgery software. The position, inclination and depth of the left maxillary incisor is carefully planned, in order not to collide with the roots of the adjacent teeth

**Fig. 6:**
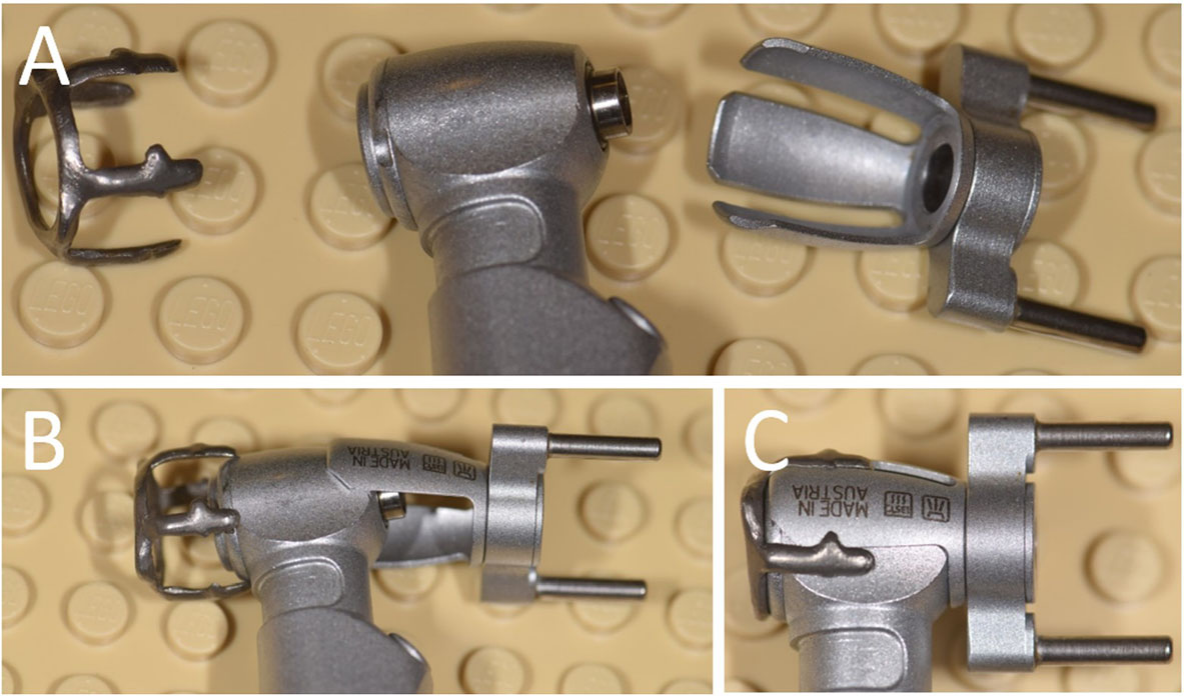
In this novel guided surgery system, the handpiece – and not the drill - is guided. **a** The adapter, the handpiece and the connector. **b** The three parts are connected; **c** The handpiece is ready for the surgery

**Fig. 7:**
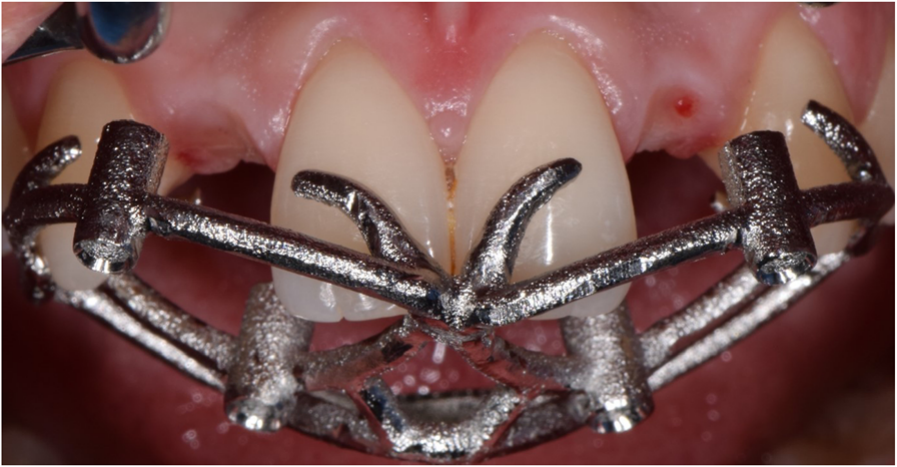
Surgery. The laser-sintered open-frame sleveless template is inserted in mouth, with excellent fit and stability

**Fig. 8:**
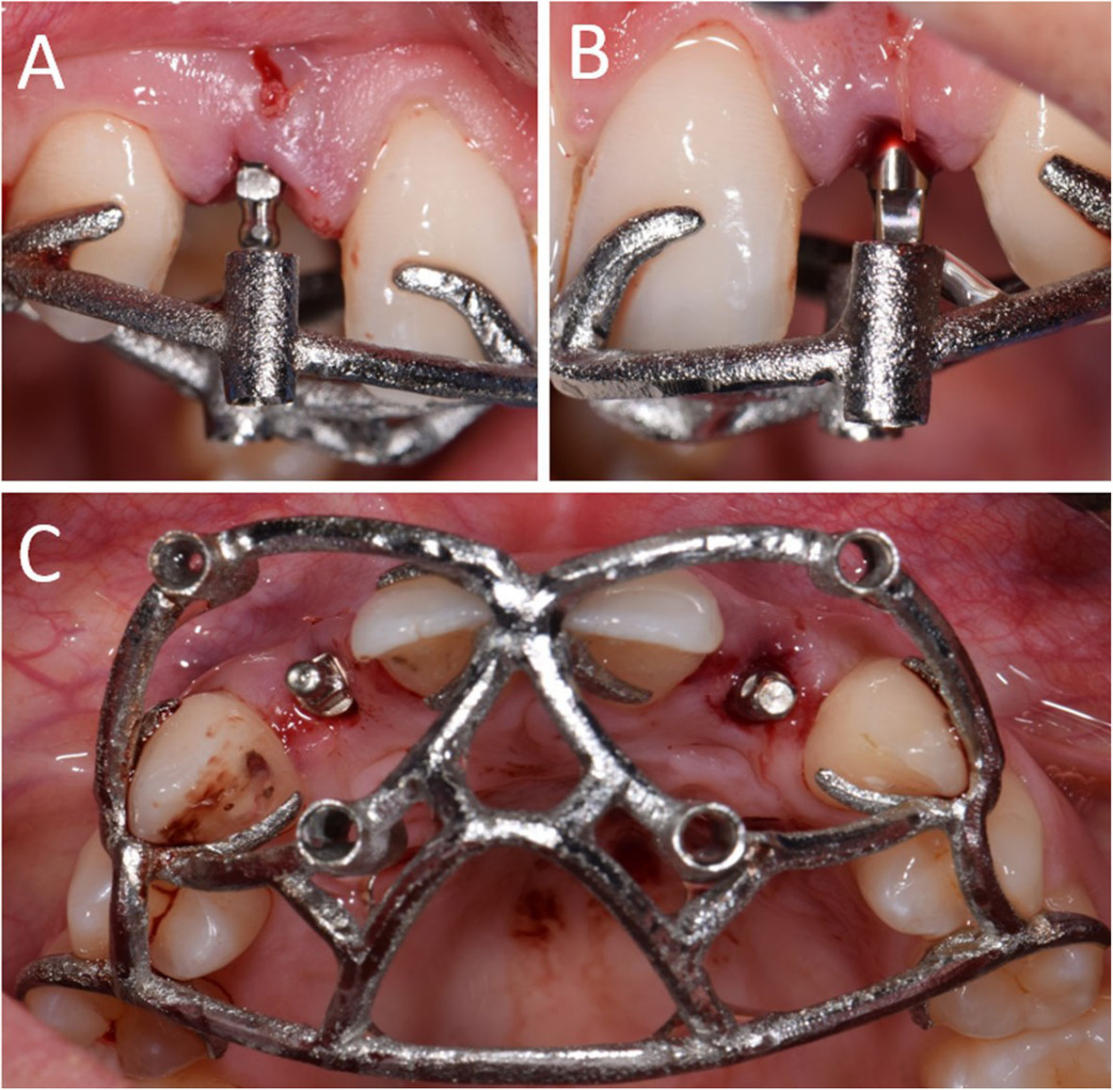
Surgery. The implants are placed through the open-frame sleveless template. **a** Placement of the right lateral incisor; **b** Placement of the left lateral incisor; **c** Both implants have been placed. Note the good visibility of the operatory field, with the guide in position

**Fig. 9:**
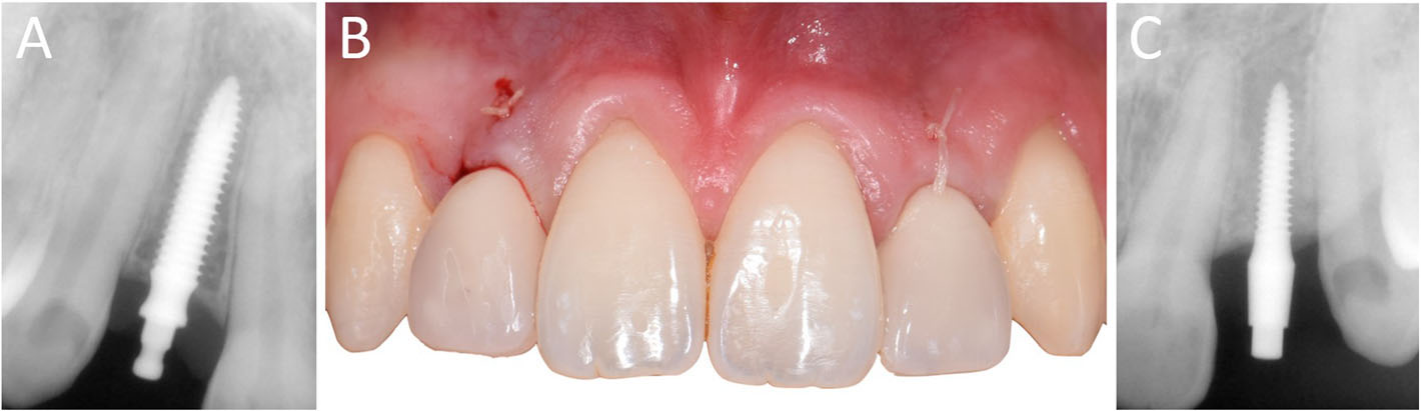
Immediate restoration. The implants are immediately restored by means of single crowns intraorally relined on temporary abutments. **a** Right lateral incisor, radiographic control immediately after implant placement; **b** Intraoral picture, frontal view of the provisionals relined on the temporary abutments immediately after implant placement; **c** Left lateral incisor, radiographic control immediately after implant placement

**Fig. 10:**
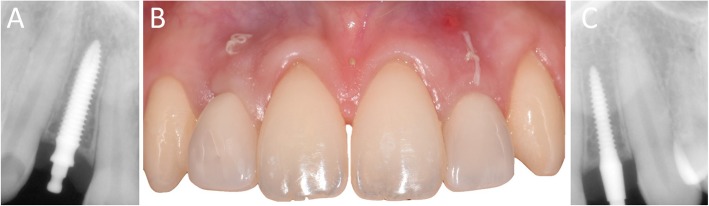
10-days post-surgical control, before sutures removal. **a** Right lateral incisor, radiographic control; **b** Frontal clinical view with provisional crowns in position; **c** Left lateral incisor, radiographic control

**Fig. 11:**
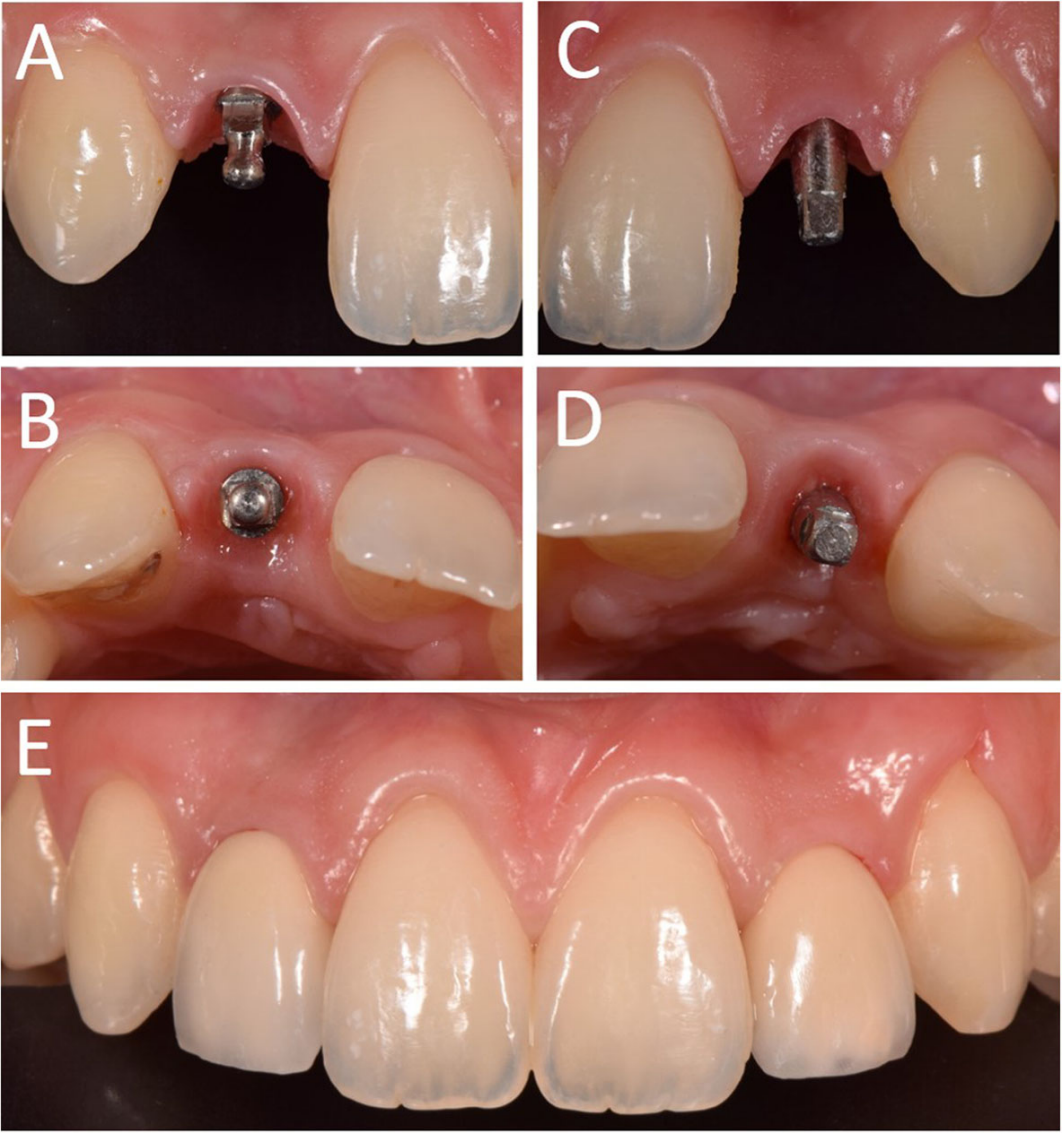
Delivery of the final restorations. **a** Right lateral incisor, frontal view of the abutment in position; **b** Right lateral incisor, occlusal view of the abutment in position; **c** Left lateral incisor, frontal view of the abutment in position; **d** Left lateral incisor, occlusal view of the abutment in position; The final single crowns were delivered and cemented on the final abutments

**Fig. 12:**
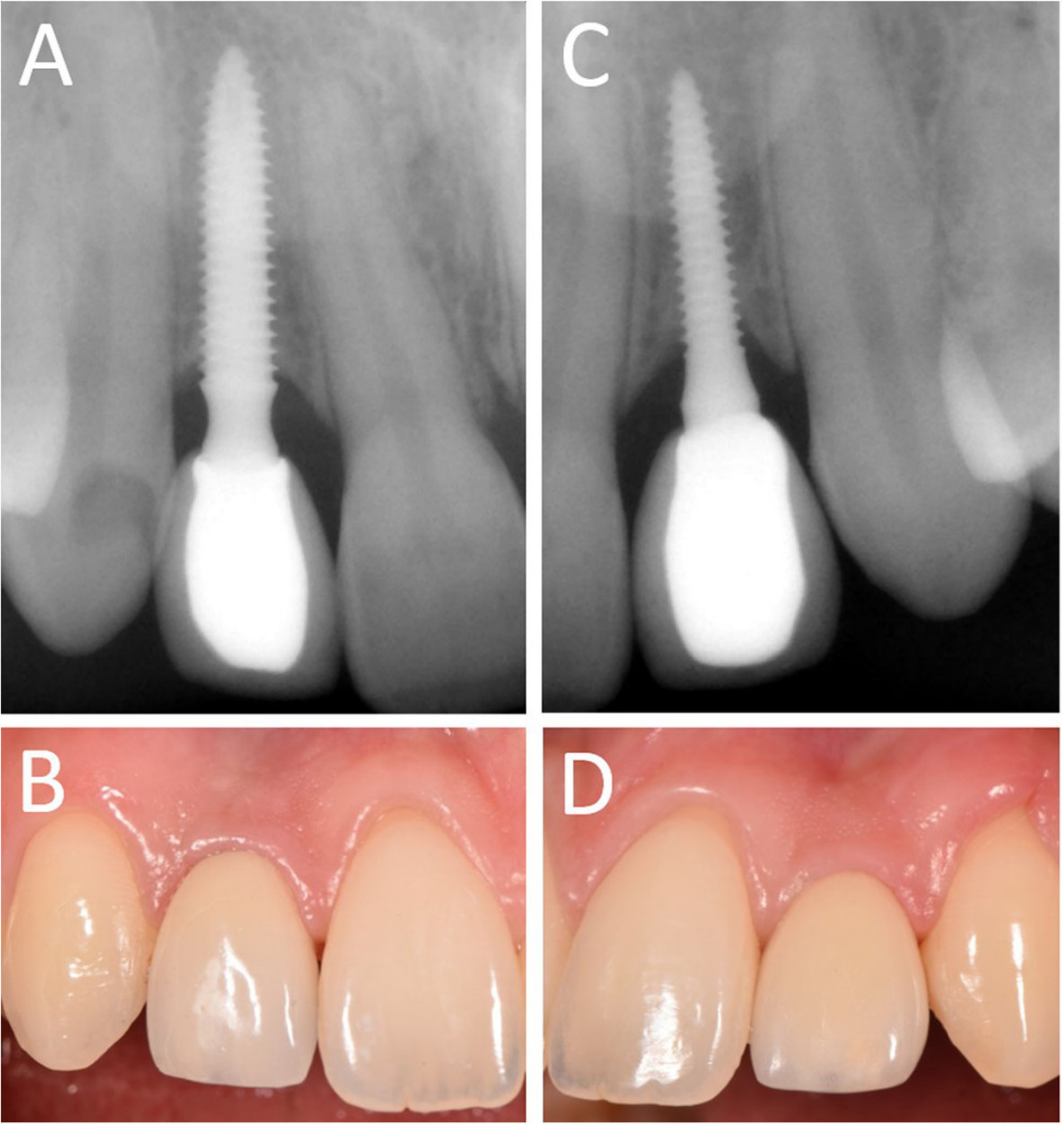
1-year follow-up control. **a** Right lateral incisor, radiographic control; **b** Right lateral incisor, frontal view. Note the soft tissues maturation; **c** Left lateral incisor, radiographic control; **d** Left lateral incisor, frontal view. Note the soft tissues stability

**Fig. 13:**
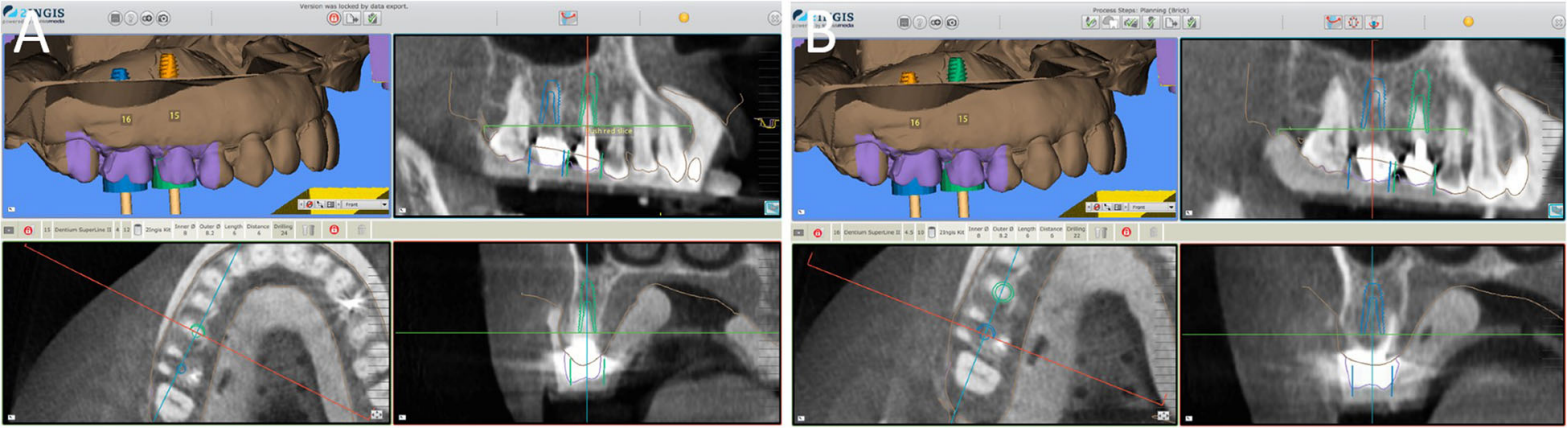
Planning of two implants in the posterior area with 2Ingis guided surgery software. **a** Second right premolar; **b** First right molar

**Fig. 14:**
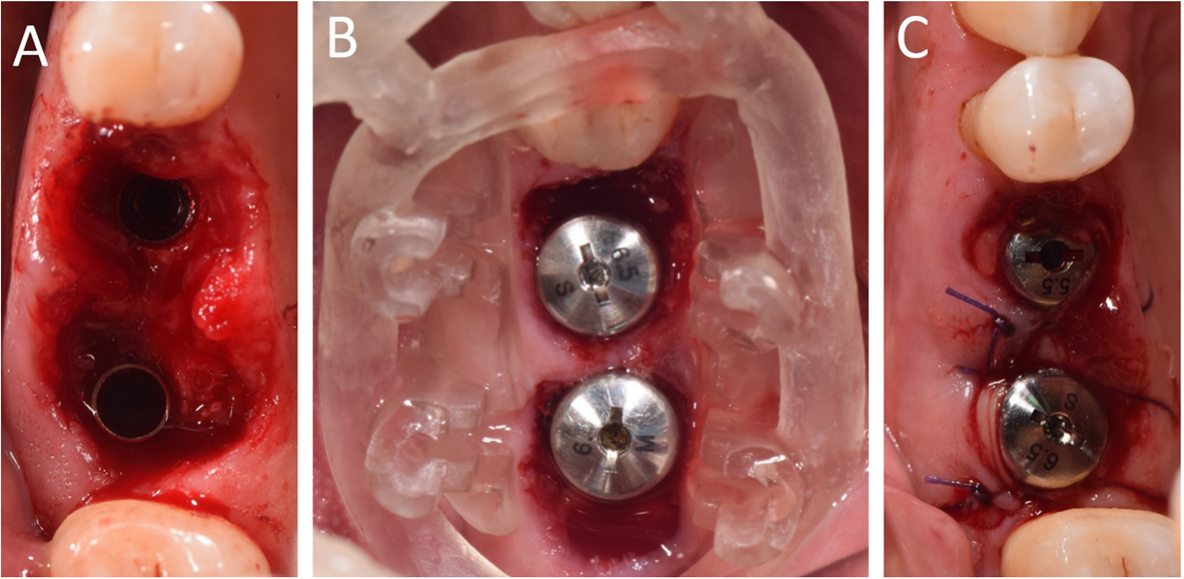
Surgery on patient. **a** The implants in position; **b** The resin guide in position after implant placement, with healing abutments already positioned; **c** Sutures

**Fig. 15:**
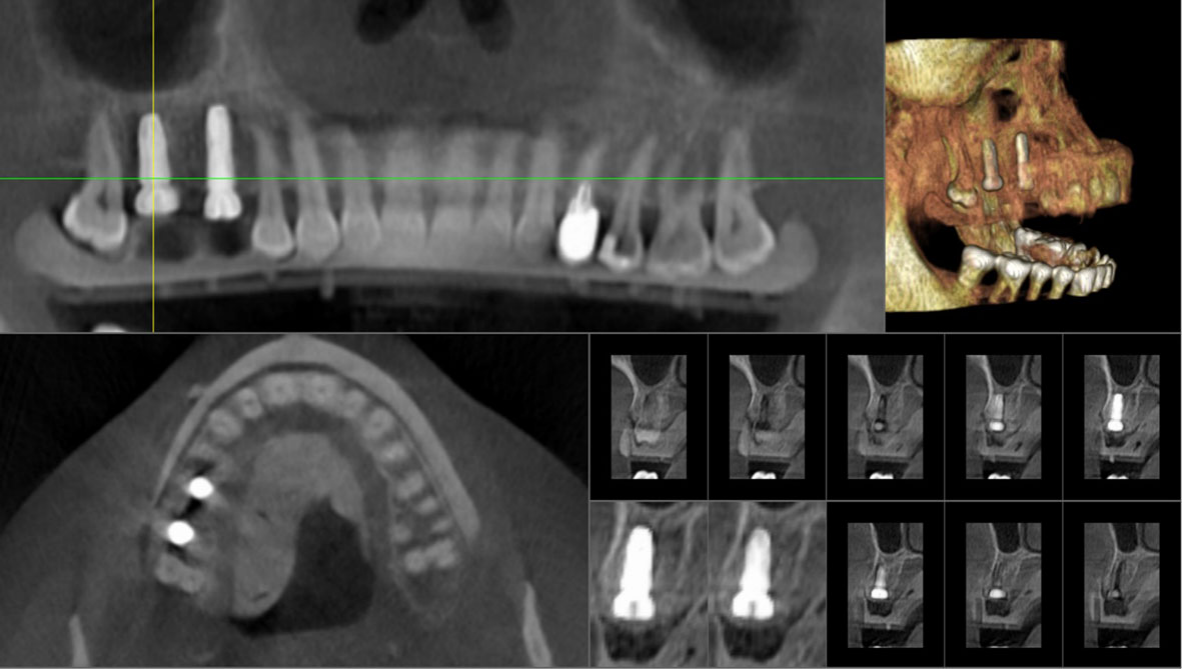
Cone beam computed tomography (CBCT) for the control of implant placement

**Fig. 16:**
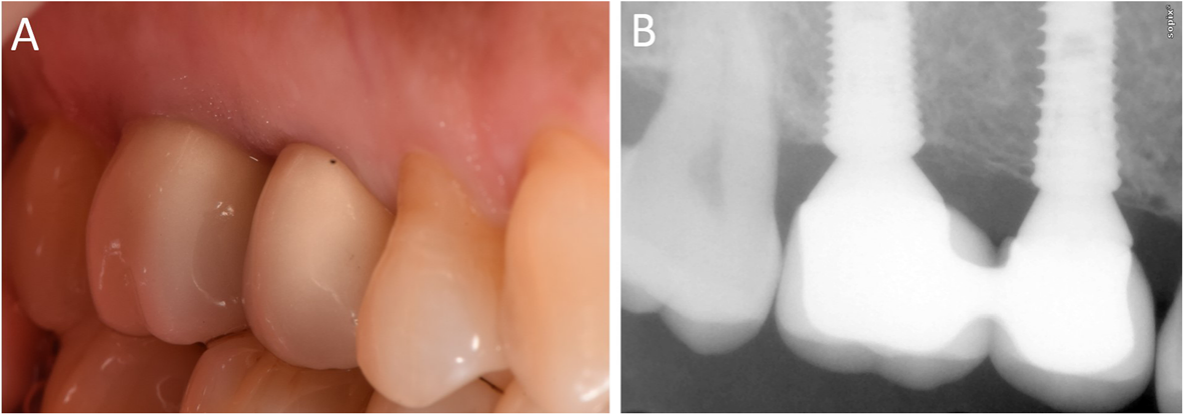
Delivery of the final restoration. **a** Clinical picture; **b** Radiographic control

**Fig. 17:**
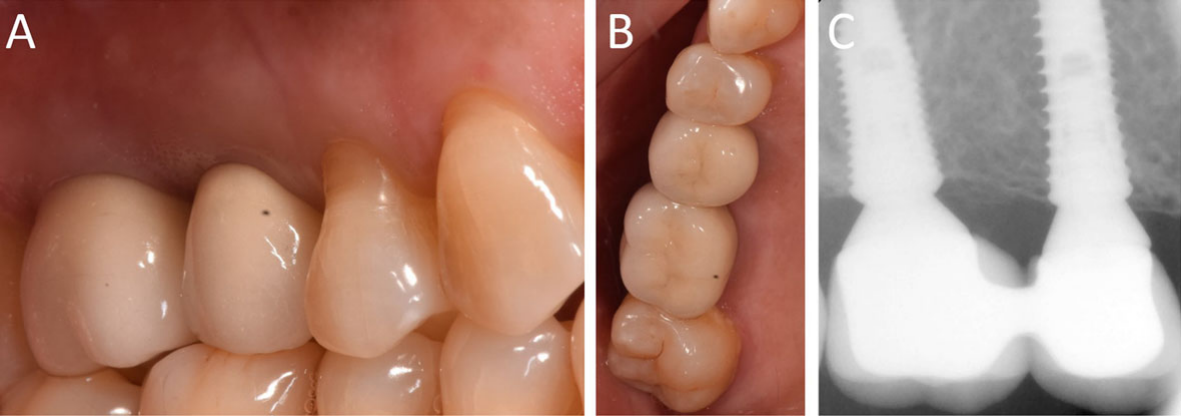
1-year follow-up control. **a** Clinical picture, lateral view; **b** Clinical picture, occlusal view; **c** Radiographic control showing stable bone levels around the implants

## Discussion

To our knowledge, this clinical study is today the only one that documents a large number of cases treated with the present new method of guided surgery, in which the handpiece is guided rather than the drills. In fact, in the literature, there are only two studies of this system for guided surgery [34, 35].

Schnutenhaus et al. tested this new sleeveless guided surgery system, in order to determine the accuracy of implant insertion with one-piece ceramic implants [34]. In total, 12 patients were enrolled in that study and installed with 20 implants by means of the aforementioned sleeveless static surgical guides [34]. The accuracy of implant placement was checked using a non-invasive method, which permitted comparison of the planning data with the actual position of the fixtures after surgery [34]. All implants were placed without any clinical problem and the mean deviations were 0.52 mm (95%CI: 0.37–0.67 mm) at the implant shoulder and 0.82 mm (95%CI: 0.56–1.08 mm) at the implant apex [34]. Finally, the mean angular deviation was 2.85° (95%CI: 2.18°-3.51°) with a deviation in height/depth of 0.35 mm (95%CI: 0.01–0.68 mm) [34]. The authors concluded that this sleeveless, open-frame, guided surgery system seems to be accurate, with little deviations between the planned and the actual position of the implants, and no clinical issues [34].

Fauroux et al. reported on 67 implants placed in 35 patients with this sleeveless, open-frame, guided surgery system [35]. These patients were treated with different protocols (one or two stage, flap or flapless, delayed or immediate loading). All cases revelead good implant placement with planning [35]. According to the authors, the main advantages with this system were the open-frame design, which allows irrigation and visual control of the surgical site, the ability to preserve the keratinized gingiva where necessary, and (being a sleeveless system) the ability to insert the implant without any contact with the sleeve [35]. The authors concluded that this system represents an interesting evolution in the field of static guided surgery [35].

The clinical results of our present retrospective study have been gratifying, and seem to confirm the evidence emerging from the previous, aforementioned studies [34, 35]. In fact, in our study, 38 patients had been treated with 110 fixtures, inserted by means of 40 sleeveless, open-frame surgical guides. Among these fixtures, 55 were inserted flaplessly, i.e., without the elevation of any surgical flap. During surgery, 34 sleeveless guides (85%) had excellent fit and stability, 4 (10%) had acceptable fit and stability, and only 2 (5%) had inadequate fit and stability for clinical use. It is important to point out that the two guides with inadequate fit and stability were made in resin. Resin guides have advantages, when compared with metal guides: they are cheaper and easier to print, and they can be more easily adapted to the site in case of minimal misfits. However, when working with resin guides, it is essential to avoid delays in the treatment, because the stability of these guides along time is not comparable to that of metal guides. In this study, in both cases in which the stability of the guides was inadequate, a delay in the treatment occurred, because patients cancelled the planned appointment for surgery. This delay may have contributed to the final, poor adaptation of the guides. The mean duration of the intervention was 23.7 min (± 6.7; median 22; 95%CI: 21.7–25.7) per template, which resulted in a mean time per implant of 6.5 min. In all patients, no immediate intra-operative complications occurred: no fractures of the surgical guides were registered, and all patients had a sufficient mouth opening to allow proceeding with surgery. Moreover, no implants were placed in an aberrant position, inclination, or depth; and no perforations of the corticals was evident nor invasion of any anatomical invalicable structure. Only a few minor immediate post-operative complications were registered, with 2 patients experiencing pain and sweeling after surgery. However, 2 implants (1.8%) were not stable after placement and had to be removed. The 108 surviving implants were restored with 36 SCs and 32 FPPs (24 two-unit bridges and 8 three-units bridges, respectively), which were followed for a period of 1 year. At the end of the follow-up period, all these restorations survived without any failure, even if a few biologic and prosthetic complications occurred. Our study therefore seems to confirm that the present system for static guided surgery is reliable and allows one to obtain clinically predictable results.

The clinical advantages of using this innovative system for guided implant surgery and this different approach to the preparation of the implant site seem to be numerous [34, 35]. First, in fact, the system presented in this study eliminates the sleeves. The use of the sleeve (metallic or not), a classic tool for guiding the drills in the vast majority of guided implant surgery systems available on the market today, has in fact some intrinsic issues [35]. The sleeve is in fact conventionally positioned above the bone site (and the overlying mucosa), which must be prepared to receive the implant; this is unavoidable if the drills are to be guided [35]. This fact, however, raises a first, intrinsic issue: it is necessary to use dedicated surgical kits with rather long drills in order to correctly prepare the surgical site [35]. In fact, the scientific literature has shown that a sleeve less than 5 mm in height is not actually able to guide the preparation of the implant site as planned (with the risk of major deviations from the original planning within the guided surgery software) [36]. If the sleeve alone “steals” at least 5 mm of space above the ridge, and this forces the clinician to use long preparation cutters, it may happen that in the posterior sectors (typically in the molar area, but also premolar) of a good percentage of partially edentulous patients, inserting implants through a surgical guide may be difficult (if not impossible) due to lack of space [3–6, 34, 35]. This is certainly one of the most clinically encountered problems with conventional guided surgery systems, and one that, to date, limits the use of these techniques in partially edentulous patients [3–6, 34, 35]. The presence of the teeth in the antagonist arch and the lack of space do not physically allow the long drills to be inserted into the surgical guide, thus rendering them unworkable. Yet, the restoration of function in partially edentulous patients is today the most frequent indication in world implantology, and it is precisely the posterior sectors that most frequently require rehabilitation with implants [37]. The innovative system for guided surgery presented in this study solves the problem of the lack of vertical space, in fact it eliminates the sleeve, and moves the guides (which are two and are inserted directly on the handpiece through a dedicated adapter) lateral to the bone crest [34, 35]. This saves space and allows the clinician to work with considerably shorter drills. The direct consequence of this is that it is also possible to work in the posterior areas of partially dentate patients, with teeth in the antagonist arch, and even with limited opening [34, 35].

But the sleeve, which is the basis of conventional guided surgery systems, does not only “steal” space vertically. It also removes space in a horizontal sense. In fact, in specific applications, e.g., the restoration of single mandibular teeth such as central or lateral incisors, the diameter of the sleeve can collide with the adjacent teeth. This creates problems during planning, which can be solved by moving the sleeve away from the adjacent tooth to avoid colliding (typical planning error that may be made by non-experienced external service operators), or by moving the sleeve higher, above adjacent teeth [3–6, 34, 35]. In the first case the implant will be positioned incorrectly, with serious aesthetic consequences. In the second case, the stolen vertical space will grow further, with the need to use even longer preparation drills, and fall into all the aforementioned problems; moreover, the literature has shown that if the distance between the sleeve and the implant site grows, the deviations grow and therefore the accuracy in positioning the implant can drastically decrease [36]. Again, driving the handpiece instead of the drill can avoid having to incur these errors. To date, there are no clinical studies on large samples of patients, comparing, in vivo, the accuracy or correspondence between the planned position in the software and the real position of the implant after the intervention, of traditional guided surgery systems versus the present, sleeveless system. However, the fact that the guides that drive the handpiece (positioned laterally) are two, could potentially help to stabilize the implant placement, reducing error [34, 35]. Certainly, then, in the partially edentulous patient, the design of the surgical templates plays a role in ensuring greater fit and therefore greater stability during surgery [6]. As shown in the literature, in fact, open surgical templates that rest selectively and for points, present an ideal stability [6] and potentially lower errors compared to closed templates that rest indiscriminately on the entire surface of the adjacent teeth [23]. In addition, the open templates allow you to check in section, on all the support teeth, the actual adaptation of the guide, and to intercept such potential errors, difficult to highlight in the classic closed templates [6, 23]. This could represent an additional advantage, increasing the accuracy of guided surgery. But the advantages of this systematic are not limited to saving of space (vertical or horizontal), and to the better fit or stability of the template. Guided implant surgery is nowadays almost exclusively conceived as a tool for placing implants flaplessly, i.e., without raising a mucoperiosteal flap [4, 5, 13–17]. This approach has some advantages, shown in the literature, but there are numerous cases in which, due to the scarce quantity of keratinized gingiva and, more importantly, due to deficiency of bone tissue, the surgeon needs to raise a flap [38]. Raising a mucoperiosteal flap allows the preservation of the keratinized gingiva (which risks being sacrificed during the operculation, in the flapless approach) by managing the soft tissues in the ideal manner [38, 39]. In the same way, it is not possible today to regenerate bone (for example, to cover exposed implant threads or to increase the bone volume by means of regenerative techniques with biomaterials or membranes) if a flapless approach is chosen. In both these scenarios, guided insertion of the implant in position, inclination, and depth remains of great utility, but it is not possible through the conventional templates for guided surgery; the sleeve (and the structure in which it is inserted) completely cover the visual and force the surgeon to work “blindly.” It is therefore not possible to manage soft tissues, nor to make small (or large) bone augmentations or perform crest splitting [40]. Once again, the sleeve creates problems for the clinician. However, if the sleeve is eliminated, and the template design is modified (open surgical guide), as happens in the system presented in this study, the surgeon can see the site on which he/she operates and consequently can better manage the soft tissues [38, 39] (for example offset the keratinized tissue, preventing it being sacrificed during the operculation) and also raise a flap with the template in place. This allows the clinician to proceed with minor and/or major bone augmentation techniques, if needed, with the guide in position.

Visibility is therefore a further, clear advantage of the method presented in this work. The presence of the sleeve is not only an obstacle to the surgeon’s vision, but also to the passage of the saline solution for the cooling of the operative site [2–6, 34, 35]. The drill to be guided is “engaged” in the sleeve, and there is no space to cool it properly while working. This represents a biological risk for subsequent implant integration: as described widely in the literature, it is important to avoid overheating of the bone during preparation of the implant site [41, 42]. The strategies to avoid it are a fluid movement during the preparation and, above all, the cooling through saline solution. Although several manufacturers have studied possible solutions to this problem, it remains evident even today, as, through conventional and closed surgical templates, it is very difficult to cool the drills [41, 42]. The guided system presented in this study definitively solves this problem: the drills are free and the cooling takes place in an optimal way, because it is the handpiece that is guided [34, 35]. Finally, a further aspect to consider is that linked to the positioning of the implant through the template. Inserting the implant through a sleeve, as in conventional guided surgery systems, can represent a biological risk; in fact, the implant surface can be contaminated, “crawling” on the walls of the sleeve [27, 28]. This risk is present if the sleeve is made of metal, and even greater in the case of resin sleeves. The risk is that particles of these materials are brought into the implant site, through the implant surface, and can interfere with the process of osseointegration [28, 29]. Since the literature has shown how the implant surface represents a key factor for survival and success [29, 43], and in consideration of the efforts made by manufacturers to produce more and more performing surfaces (i.e., able to accelerate the processes of bone healing), it is unforgivable to risk compromising everything by contaminating the fixtures with external materials. The guided surgery system presented in this clinical study solves this problem, because the sleeve is eliminated and the implant is inserted through the handpiece: it is, in other words, free from contact with other, undesired surfaces [34, 35].

Despite the clinical success reported in this study and the advantages given by this modern approach to guided surgery, it should be noted that today there is insufficient data on the accuracy of the present system [34], compared to conventional systematics. In other words, we do not have sufficient mathematical data on the system; further studies will be needed in that direction. In addition to this, the present study has a retrospective design and is based on the case histories of a single, experienced operator; for this reason, this study does not allow definitive conclusions on the validity of this new system. Moreover, multiple implant systems have been used here, and the guides were printed with two different materials (25 of them in metal, 15 in resin). These can be considered as further limitations of this study. Prospective and multicenter studies, involving dental centers and operators with different levels of experience, will be necessary to dispel any doubt about the reliability of this system.

## Conclusions

This retrospective clinical study presented results with a novel guided surgery system with a sleeveless, open-frame structure, in which the surgical handpiece (not the drills used for preparation) is guided. In total, 38 patients who had been treated with 110 implants inserted by means of 40 sleeveless, open-frame guides were examined. With regard to surgery, the fit and stability of almost all open-frame sleeveless guides (36/38) was adequate, and only 2 guides were not suitable for clinical use. The mean duration of the intervention was 23.7 min (± 6.7). Immediately after placement, 2 fixtures were not stable and had to be removed. The 108 surviving implants were restored with 36 single crowns and 32 fixed partial prostheses, that survived until the 1-year follow-up, with a low incidence of complications. Although this clinical study has limits (limited patient sample, retrospective design, single operator, and no evaluation of the accuracy of the implant placement), the novel guided surgery system with sleeveless, open frame–structure guides presented here seems to be clinically safe and reliable. Obviously, multicenter studies on a larger sample of patients involving different operators, and evaluating the accuracy in implant position, are needed to dispel any doubt about the reliability of this system.

## Data Availability

The clinical and radiographic documentation of the patients enrolled in this study, as well as the surgical and prosthetic planning data belong to the authors, and are available only upon reasonable request, after approval by all the authors.

## Abbreviations

3D: Three-dimensional
CAD: Computer-assisted-design
CBCT: Cone beam computed tomography
CI: Confidence intervals
DICOM: Digital imaging and communication in dentistry
FPP: Fixed partial prosthesis
SC: Single crown
SD: Standard deviation
